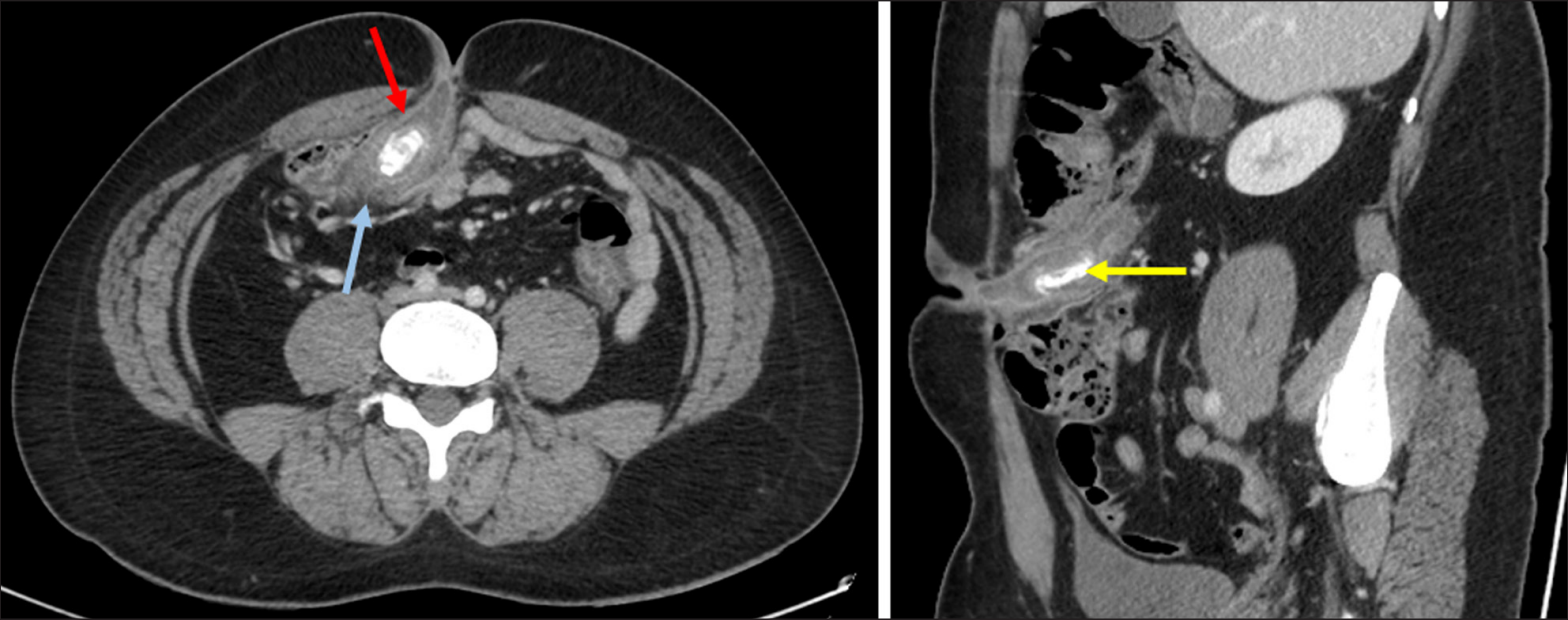# A Rare Complication of Appendicostomy

**DOI:** 10.5334/jbsr.2753

**Published:** 2022-05-04

**Authors:** Romain Lacrosse, Cristina Dragean

**Affiliations:** 1 Université Catholique de Louvain, BE; 2 Cliniques Universitaires Saint-Luc, BE

**Keywords:** Malone appendicostomy, appendicolith, antegrade continence enema

## Abstract

**Teaching Point:** The acute appendicitis in a context of Malone appendicostomy complication is very rare but can occur and may be challenging to diagnose due to its unusual position.

## Case History

A 33-year-old male patient with a history of Malone appendicostomy (MA) for neonatal ano-rectal malformation presented with fever (40°C), increasing periumbilical pain, diarrhoea and nausea. Clinical examination revealed periumbilical cutaneous inflammation. Bloods showed only discrete increase of CRP. Abdominal computed tomography (CT) examination revealed wall thickening (***Figure 1***, red arrow axial image) and enlargement of the appendicostomy due to a large 30 × 10 mm appendicolith (***Figure 1***, yellow arrow sagittal oblique image) with discrete fat stranding (***Figure 1***, blue arrow axial image) and local reactional mesenteric lymph nodes. No bowel perforation was detected. The retained diagnosis was acute appendicitis, and the patient was treated conservatively with a satisfactory clinical evolution. Years later, relapsing symptoms led to surgical ablation of the appendicostomy. MA anastomoses the appendix to the deep part of the umbilicus and includes a valve mechanism allowing for antegrade colonic enema flushing without stool leakage in patients with fecal incontinence. Frequent complications of MA include stomal orifice stenosis as well as stomal infection and appendiceal perforation during catheterization [1]. However, the presence of inflammation of the appendicostomy secondary to obstruction is rare and may be challenging to diagnose radiologically due to the unusual position of the appendix.

**Figure 1:**